# Cost-effectiveness of HLA-DQB1/HLA-B pharmacogenetic-guided treatment and blood monitoring in US patients taking clozapine

**DOI:** 10.1038/s41397-017-0004-2

**Published:** 2018-01-03

**Authors:** François R. Girardin, Antoine Poncet, Arnaud Perrier, Nathalie Vernaz, Mark Pletscher, Caroline F. Samer, Jeffrey A. Lieberman, Jean Villard

**Affiliations:** 1 Division of Clinical Pharmacology and Toxicology, Department of Anesthesiology, Intensive Care, and Clinical Pharmacology, University Hospitals of Geneva and University of Geneva, Geneva, Switzerland; 2 Medical Direction, University Hospitals of Geneva and University of Geneva, Geneva, Switzerland; 3 The Clinical Research Centre, Division of Clinical Epidemiology, Department of Health and Community Medicine, University of Geneva and University Hospitals of Geneva, Geneva, Switzerland; 4 Department of Internal Medicine, Rehabilitation and Geriatrics, Geneva University Hospitals and Geneva Faculty of Medicine, Geneva, Switzerland; 5 Finance Direction, University Hospitals and University of Geneva, 1205 Geneva, Switzerland; 6 Winterthur Institute of Health Economics, Zurich University of Applied Sciences, Winterthur, Switzerland; 7 Department of Psychiatry, Columbia University and New York State Psychiatric Institute, New York, NY 10032 USA; 8 Division of Nephrology, University Hospitals of Geneva and University of Geneva, Geneva, Switzerland

**Keywords:** Health policy, Health services

## Abstract

Less than 1% of adult patients with schizophrenia taking clozapine develop agranulocytosis, and most of these cases occur within the first weeks of treatment. The human leukocyte antigen (HLA) region has been associated with genetic susceptibility to clozapine-induced agranulocytosis (single amino acid changes in HLA-DQB1 (126Q) and HLA-B (158T)). The current study aimed to evaluate the cost-effectiveness, from a healthcare provider’s perspective, of an HLA genotype-guided approach in patients with treatment-resistant schizophrenia who were taking clozapine and to compare the results with the current absolute neutrophil count monitoring (ANCM) schemes used in the USA. A semi-Markovian model was developed to simulate the progress of a cohort of adult men and women who received clozapine as a third-line antipsychotic medication. We compared current practices using two genotype-guided strategies: (1) HLA genotyping followed by clozapine, with ANCM only for patients who tested positive for one or both alleles (genotype-guided blood sampling); (2) HLA genotyping followed by clozapine for low-risk patients and alternative antipsychotics for patients who tested positive (clozapine substitution scheme). Up to a decision threshold of $3.9 million per quality-adjusted life-year (90-fold the US gross domestic product per capita), the base-case results indicate that compared with current ANCM, genotype-guided blood sampling prior to clozapine initiation appeared cost-effective for targeted blood monitoring only in patients with HLA susceptibility alleles. Sensitivity analysis demonstrated that at a cost of genotype testing of up to USD700, HLA genotype-guided blood monitoring remained a cost-effective strategy compared with either current ANCM or clozapine substitution.

## Introduction

Treatment-resistant schizophrenia (TRS) is a highly disabling psychiatric disease that affects approximately one-third of psychotic patients over the course of their illness [1]. Clozapine is an atypical antipsychotic that is available as a generic drug, has significant therapeutic efficacy, and produces desirable quality-of-life outcomes [2]. These benefits must be weighed against potential hematologic or metabolic adverse effects, and a network meta-analysis found little evidence of superior efficacy of clozapine relative to other second-generation antipsychotics in subgroups of patients with TRS [1]. Clozapine treatment remains restricted to a small proportion of patients with TRS who did not previously improve after treatment with two or more antipsychotics [3]. A primary reason for the limited use of clozapine is the potential risk for severe clozapine-induced agranulocytosis (CIA), a blood dyscrasia affecting polymorphonuclear leukocytes; additionally, the associated long-term blood monitoring burden deters clinicians and patients from clozapine therapy [4]. However, approximately 80% of CIA cases occur within 18 weeks of the introduction of clozapine; [5] after one year of clozapine therapy, the incidence of CIA decreases to 0.07% or less [6]. Nonetheless, clozapine administration remains subject to long-term absolute neutrophil count monitoring (ANCM) and, in certain countries (e.g., the US, the UK, and Australia), to registry-based prescribing systems. ANCM schedules differ significantly across countries, and their utility has been debated [7]. After the first six months of clozapine treatment, the Netherlands Clozapine Collaboration Group permits a reduction in monitoring frequency to four times per year [8]. In the US, where the requirements are stringent, weekly ANCM is maintained during the first six months of treatment (weeks 1–26), followed by ANCM every two weeks (weeks 26–52) and monthly ANCM after one year of treatment. In most European countries, ANCM is performed throughout the first 18 weeks, followed by monthly monitoring. In 2015, the US Food and Drug Administration (FDA) approved a new shared risk evaluation and mitigation strategy (REMS) to provide guidance for all medicines containing clozapine [3].

In the 1990s, the human leukocyte antigen (HLA) region was found to be associated with genetic susceptibility for CIA [9]. In 2014, a novel genome survey using whole-exome sequencing and genome-wide genotyping indicated that CIA was associated with single amino acid changes in HLA-DQB1 (126Q) and HLA-B (158T) [10]. After more than 25 years of research into genetic factors, a genetic predictor test with wide clinical application has still not been developed [11]. Despite limited test sensitivity [12], we hypothesized that pharmacogenetic-guided treatment based on germline DNA is cost-effective because it identifies individuals at higher risk for agranulocytosis and reduces the ANCM burden for patients who lack susceptibility alleles.

## Materials and methods

### Overview of the decision analytic model

**Fig. 1:**
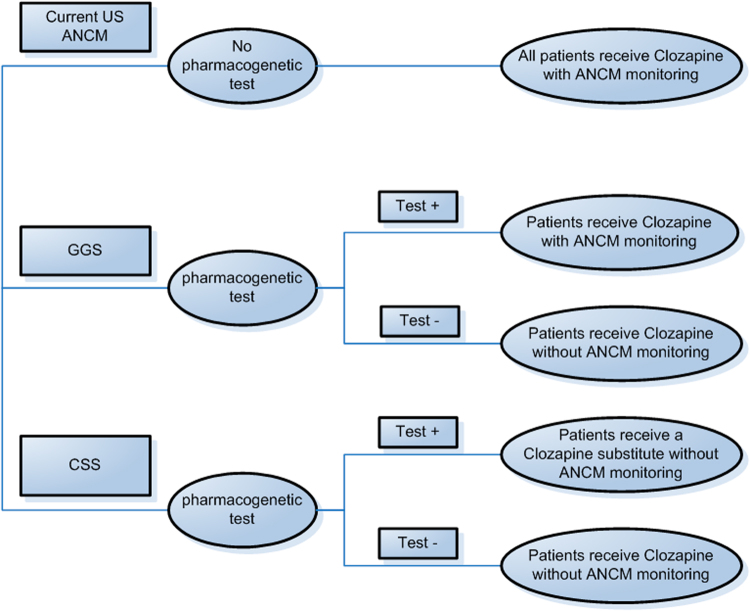
Decision tree for the compared strategies. The current US ANCM system was compared to two alternative strategies: (1) genotype-guided sampling (GGS) and (2) a clozapine substitution scheme (CSS)

To investigate whether the current US intensive blood monitoring might be restricted to high-risk individuals given new shared REMS in 2015, we developed a decision-analytical model to compare the current US ANCM scheme with two pharmacogenetically based schedules. We hypothesized that HLA genotyping was performed prior to clozapine initiation and conditioned the monitoring schedules. Two alternative schemes to current ANCM were defined: (I) clozapine for all patients. Targeted ANCM only in patients testing positive for one or both susceptibility alleles (genotype-guided sampling (GGS)), and (II) clozapine for patients testing negative for both susceptibility alleles plus an antipsychotic substitute for patients testing positive for one or both susceptibility alleles. No ANCM for any patients (clozapine substitution scheme (CSS)) (Fig. 1).

We hypothesized that: (I) GGS could be cost-effective because the reduced costs may offset the marginal effectiveness by removing long-term ANCM in lower-risk patients who tested negative for the HLA alleles; and that: (II) CSS could be cost-effective because it completely removes the costly ANCM due to switching from clozapine to an antipsychotic substitute in patients with a higher risk (incidence of CIA 1.8% instead of 0.7%), producing less follow-up burden.

### Model cohort

For the decision-analytical model, we developed a semi-Markovian framework allowing for probabilistic approaches and performed the cost-effectiveness analysis based on the US monitoring schedule and both abovementioned pharmacogenetic alternatives (see decision tree, Fig. 1).

The study population consisted of adult men and women with TRS who received clozapine as a third-line antipsychotic medication. To maintain consistency with the analytical decision model, we specifically considered agranulocytosis cases (defined as cases involving an absolute neutrophil count lower than 0.5 × 10^9^ per liter, with high sepsis risk); in contrast, the authors of a CIA Consortium publication [10] also considered the risk of granulocytopenia. The allele prevalence, probabilities of confirmed CIA among positive patients treated with clozapine, and the sensitivity and specificity of the HLA-DQB1 and HLA-B testing in TRS patients treated with clozapine were calculated from genome-wide genotyping and a whole-exome sequencing study. The genotyping sensitivity was 0.41, and the specificity was 0.85, which were calculated on the basis of a total of 301 samples from the CIA Consortium (39 patients of the 95 agranulocytosis cases presented one or both of the HLA-DQB1 or HLA-B alleles, and 175 among 206 treated control cases taking clozapine presented no allele variants, genotyped on the Illumina OmniExpress array at Duke University, North Carolina, USA).

The outcomes included mortality, mean cost per patient, and mean quality-adjusted life years (QALYs) per patient over a 3-year period, which was employed because scant long-term estimates from registry-based prescribing systems are available beyond this follow-up duration. In accordance with the recommendations of the US Panel on Cost-Effectiveness in Health and Medicine [13] and to provide uniformity in cost-effectiveness analyses to permit comparisons across studies, we reported incremental cost-effectiveness ratios (ICERs) for alternative schedules based on HLA-DQB1 and HLA-B allele testing. These ratios were compared with the current US ANCM as the reference strategy and represented the probability of maximum cost-effectiveness for each strategy, depicted as cost-effectiveness acceptability curves (CEACs) [14]. The decision threshold value for one additional QALY indicated which strategy was the most likely to maximize health benefits given limited resources. Consistent with recent US considerations, a $50,000 per QALY threshold is a commonly accepted benchmark for the value of care [15].

### Model structure, simulations, and transition probabilities

We developed additional functions to integrate genotype-based schedules into a validated semi-Markovian model structure [16] with time-dependent transition probabilities between health states (HS) to simulate cohorts of TRS patients on clozapine. These cohorts included 100,000 patients who may or may not have been previously genotyped for HLA alleles and who continuously transitioned through the four HS. Briefly, individuals with TRS start in the state “schizophrenia treated by clozapine” and could continuously take clozapine without CIA and remain in the same HS. CIA was defined as an absolute neutrophil count below 0.5 × 10^9^ per liter with potential infection, and, in some cases, sepsis-related death. Patients could be switched to an alternative antipsychotic for reasons unrelated to CIA (e.g., weight gain, anticholinergic side-effects, and further adverse events) or die for other reasons unrelated to CIA-induced sepsis. The model was developed using the R^®^ programming software package (R Foundation for statistical computing, Vienna, Austria, Version 2.15.2). Further details on model structure can be found elsewhere [16].

### Cost estimates and utilities

The cost-effectiveness analysis was conducted from a third-party payer perspective using direct medical expenditures (see Supplementary Table 1). The mean expected costs of HLA-DQB1 and HLA-B testing was set at $200 based on reimbursement codes used by histocompatibility laboratories. To derive country-based estimates from the clinical situations of hospitalized patients with complicated drug-induced infections, sepsis costs were calculated based on medical statistics using Swiss Diagnosis Related Groups (DRG) tariff rates and corrected for inflation (see Supplementary Table 1). Cases of sepsis due to a drug-induced severe neutropenia or agranulocytosis were identified by an ICD-10-GM main diagnosis of sepsis (A39.2, A40, A41, R65) or by substance-induced neutropenia (D70.10-D70.19). Fifty-one cases fulfilled these inclusion criteria in the medical statistics database. The average length of stay was 19.4 days, and the average cost per inpatient stay was $31,398 in 2013 (Table 1). These cost calculations are consistent with a US hospital-related sepsis cost of $32,421 (interquartile range $20,745–40,835) [17] and have been widely tested in sensitivity analyses. All cost results are in US currency with a rounded exchange rate of US $1.00 = 1.00 Swiss franc (March 20th, 2017). Antipsychotic drug costs were based on US average wholesale prices from a manufacturing directory resource (http://www.redbook.com/redbook/online) [18], and clozapine dose equivalences were calculated according to the daily defined dose (DDD) for alternative follow-on medication as described previously [19]. We converted clozapine doses to doses of antipsychotic substitutes using the DDD. The following antipsychotics were assumed to be clozapine substitutes: amisulpride, asenapine, aripiprazole, chlorpromazine, flupentixol, haloperidol, lurasidone, olanzapine, paliperidone, promazine, quetiapine, risperidone, sertindole, and ziprasidone.

**Table 1:** Key input parameters

	Parameter estimates	Probabilistic sensitivity analysis	Sensitivity analysis
Genetic test			
Sensitivity	0.41	Yes	0.27–0.54
Specificity	0.85	Yes	0.80–0.90
Cost (US$)	$200	Yes	0–1 000
Sepsis cost (US$)	$31,398	Yes	5000–50,000

Quality-of-life variations across the model were applied to each time period spent in HS, weighted by TRS utilities [20], and derived from the Euro-QOL-5-Dimensions (EQ-5D) questionnaire estimates from the Medical Expenditure Panel Survey [21]. Additional estimates for costs and utilities with references are available in Supplementary Table 1. Discount rates of 3% were applied to QALYs and costs [22].

### Sensitivity analyses

We conducted one-way sensitivity analyses to assess the robustness of the model outcomes by varying key parameters driving the model throughout credible ranges: sensitivity and specificity of genetic testing, risk of CIA, rate of severe infection in patients with agranulocytosis, case-fatality of sepsis in patients with agranulocytosis, utility of severe infectious episodes, TRS treated by clozapine, TRS treated by clozapine substitute, costs of genetic testing, clozapine substitutes, ANCM, sepsis management, and discount rates on costs and QALYs.

We performed probabilistic sensitivity analyses to explore joint parameter uncertainty and whether parameter variability is translated into outcome variability. Parameter values were drawn at random from the assigned distributions for each of the 10,000 simulated cohorts, and 95% confidence intervals were obtained according to the 0.025 and 0.975 percentiles of the simulated results (for assigned distributions, see Supplementary Table 2).

## Results

Using an agranulocytosis incidence of 0.7% and test sensitivity of 0.41, the number of patients needed for genotyping was calculated as follows: (100 / (0.7 × 0.41)). In all, 348 screened patients were needed to prevent one case of agranulocytosis. This estimate approximates a previous estimation with higher agranulocytosis prevalence but with a lower, single HLA-DQB1 genotyping sensitivity [23].

In a cohort of 100,000 TRS patients taking clozapine, the 3-year all-cause mortality was 1.70% for the current US monitoring schedule, 1.71% for the GGS, and 1.78% for the CSS. The monitoring number required to avoid one death was nearly 10,000 patients in the current US ANCM scheme when compared with that of the GGS. Considering a 3-year period, the mean survival time adjusted for quality of life was 669.9 quality-adjusted life-days (QALD) for current US ANCM and 669.8 QALD for the GGS (−0.1), compared to 660.2 QALD for the CSS (−9.7). The mean patient cost was $13,694 in the current US ANCM, $13,091 in the GGS (-$603), and $13 738 (+$44) in the CSS (Table 2). The lower costs of the GGS strategy were associated with the targeted monitoring only in patients with alleles of susceptibility for the development of a CIA episode because the GGS strategy limited resource use over a longer period of time and offset initial genetic testing expenses. In the CSS, the resource savings of restricted ANCM did not recover the extra costs generated by clozapine substitutes, which are more expensive and less effective than clozapine.

**Table 2:** Base-case scenario results

Outcomes	Current US strategy	Genetically guided strategy	Clozapine substitution strategy
Cumulative mortality (%)	1.70 (1.48–1.92)	1.71 (1.49–1.93)	1.78 (1.54–2.02)
Mean survival time per patient adjusted for quality of life (quality-adjusted life-days)	669.9 (618.4–719.1)	669.8 (618.3–719.0)	660.2 (603.7–713.7)
Cost per patient (US$)			
Total	$13,694 (7752–19,626)	$13,091 (7154–19,023)	$13,738 (7240–20210)
Sepsis	$98 (60–140)	$163 (100–230)	$123 (73–180)
Clozapine treatment	$1453 (1442–1464)	$1453 (1442–1464)	$1234 (1157–1301)
Substitute treatment	$11 122 (5189–17,057)	$11,120 (5188–17,052)	$12,181 (5671–18,648)
ANCM	$1021 (1015–1027)	$154 (108–207)	0
Genetic testing	0	$200	$200
ICER (million US$ per QALY)^a^	$3.93 (2.01–8.17)		Dominated

Data in brackets show 95% CIs from probabilistic analyses

^a^The genetically guided strategy was the reference strategy

**Fig. 2:**
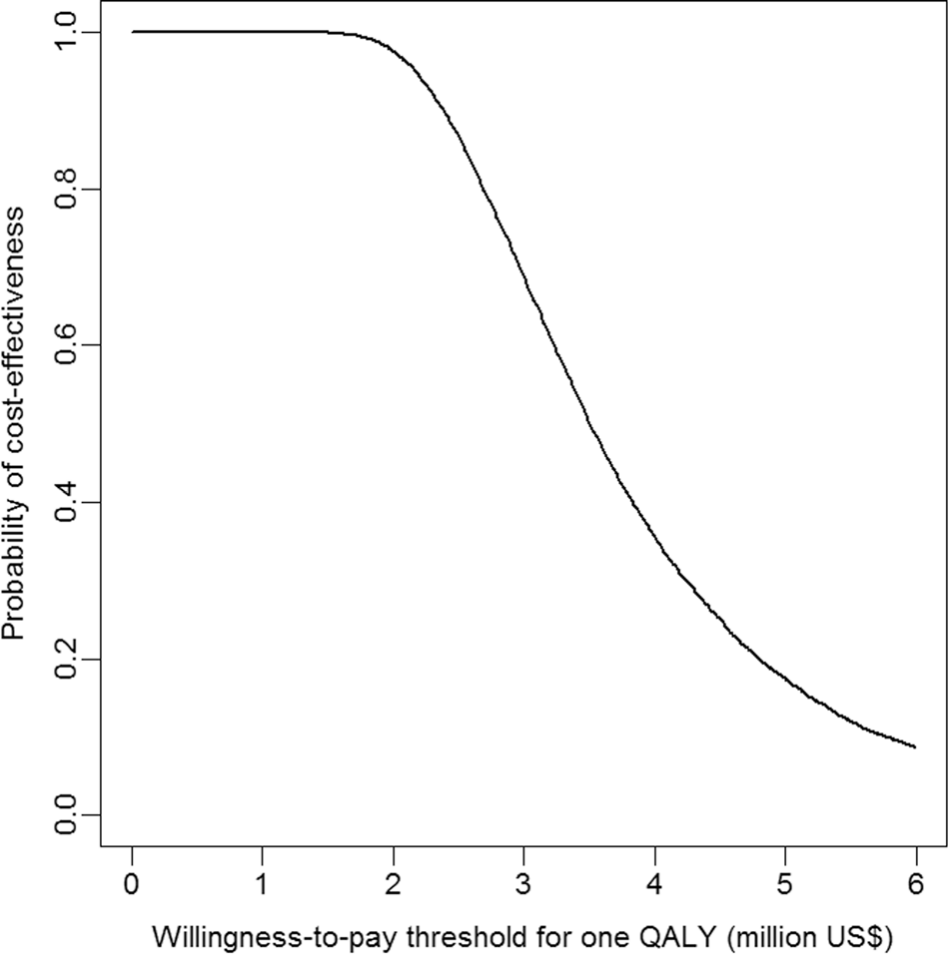
Cost-effectiveness acceptability curve to compare alternative GGS with the current blood monitoring schedule

Compared with the current US ANCM schedule, the CSS was the least effective but also a less costly option in the majority of Monte Carlo simulations (see red cloud, Supplementary Fig. 1); the CSS was not a cost-effective strategy below the willingness-to-pay (WTP) threshold of $3 million (results not shown). GGS appeared to be the least expensive option in the majority of the Monte Carlo outputs with a marginal utility loss (<0.1 QALD) compared with the current US ANCM (see green cloud, Supplementary Fig. 1). The scatter plots for simulations were concentrated in the southwest quadrant of the cost-effectiveness plan, indicating that the GGS had a high probability of being the most cost-effective strategy until a WTP of $3 million was reached (Fig. 2). Relative to the GGS, the CSS was a dominated strategy (i.e., a more costly and less effective strategy). Subsequently, the results for the GGS were compared with those for the current US ANCM scheme.

The ICER for the GGS compared with the current US ANCM scheme was $3.93 million per QALY, meaning the current schedule entails an extra cost of $3.93 million (95% CI: 2.01–8.17) to save one QALY compared with the GGS (Table 2).

In the one-way sensitivity analysis, we checked the model robustness by varying key input parameters (Supplementary Table 3). The cumulative mortality and mean survival times (adjusted and unadjusted for quality of life) remained virtually unchanged when the risk of agranulocytosis varied from 0.38% to 2.0%, the infection-related mortality varied from 2.5% to 20%, and the prevalence of variant alleles varied from 10% (sensitivity of 27% and specificity of 90%) to 20% (sensitivity of 54% and specificity of 80%). The QALYs were also unchanged when the utilities varied from 0.1 to 0.6 after a sepsis episode. The tests for mean costs per patient were marginally affected by varying the risk of agranulocytosis, infection-related mortality, and the accuracy of HLA allele genotyping.

**Fig. 3:**
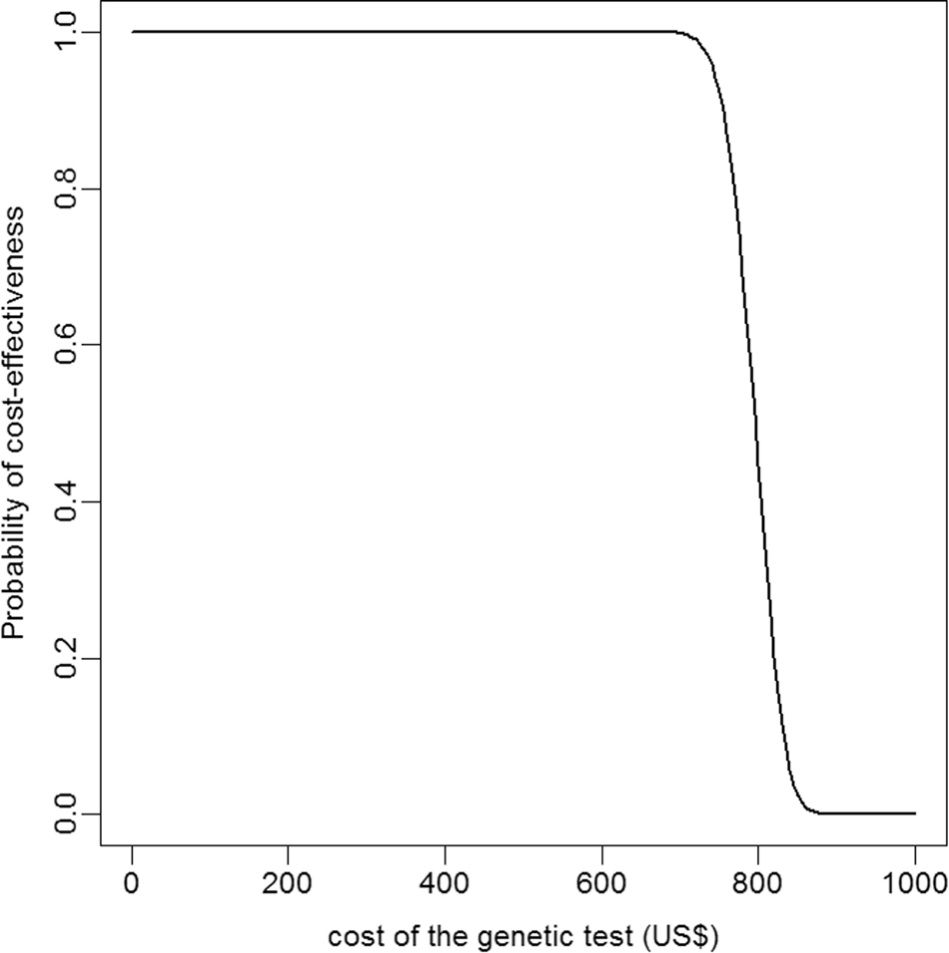
Probability of GGS being cost-effective according to testing costs and given a willing-to-pay threshold of $50,000

By contrast, the ICER was sensitive to the risk of agranulocytosis, the performance and costs of pharmacogenetic testing, the infection-related mortality, the price of clozapine alternatives, and, to a lesser extent, the QOL when less effective clozapine substitutes were prescribed. Incremental costs clustered between $500 and $700, in favor of the GGS (see Supplementary Fig. 1), suggesting that if the pharmacogenetic testing cost estimate was higher than $1000, the GGS would be dominated by the current US ANCM. Fig. 3 represents the cost-effectiveness probability of the GGS for a $50,000 WTP according to pharmacogenetic test costs. At this WTP, the GGS had a probability of one to be cost-effective when the cost of the genetic test was less than $700. However, the cost-effectiveness dropped sharply when the genetic test was more than $800. These results were similar for a WTP of $100,000 (results not shown).

## Discussion

To our knowledge, this study is the first comparative cost-effectiveness analysis of US ANCM requirements using two alternative strategies based on pharmacogenetic testing of two single HLA alleles. We considered the HLA-DQB1 and HLA-B alleles in schizophrenic patients treated with clozapine to prevent agranulocytosis either by restricting ANCM to patients with the alleles of susceptibility (GGS) or by foregoing ANCM in all patients and selecting an alternative antipsychotic drug instead for patients with HLA alleles of susceptibility (CSS strategy). The results indicated the GGS was cost-effective to a WTP threshold of $3.9 million per QALY, although the results were sensitive to changes in the costs of genetic testing, CIA prevalence, and infection-related death rates.

Although translation processes and clinical implementation of pharmacogenomics, including proof of cost-effectiveness, have been emphasized [24], these results expand current knowledge for the Choosing Wisely campaign [25] and may encourage physicians to improve the efficiency of health care when applying empirical procedures. In 2015, the FDA made changes to the requirements for monitoring, prescribing, and dispensing clozapine in line with the recent REMS guidance (and replacing the six existing clozapine registries). Other important changes include prescribing clozapine for patients with benign neutropenia, as well as using specific ANCM algorithms for these patients and those who previously benefited from clozapine but developed severe neutropenia or agranulocytosis.

The relationship between CIA risk and the proportion of patients in the group with variant HLA alleles has been recently studied [26, 27]. Given the low incidence of CIA, to be clinically applicable, HLA allele testing must have a sensitivity of approximately 50%. Therefore, the testing of the HLA-DQB1 allele alone (sensitivity 21.5%; specificity 98.4%) [28] was not accurate enough to merit consideration. Combining HLA-DQB1 with HLA-B epitope testing increased the sensitivity for CIA prediction to 41% (CI 95%: 31–51%) and showed a 30% reduction in the lower risk group (a reduction in CIA incidence from 0.7% to <0.5%), which approximates the agranulocytosis risk of other antipsychotics [6]. Although the sensitivity value of the pharmacogenetic test is currently not sufficiently high for clinical application, it is nonetheless cost-effective because the ICER of current US ANCM schedules remains largely above conventional WTP thresholds (i.e., $50,000 per QALY). The use of a Health Technology Assessment with decision thresholds represents societal preferences regarding disinvestment and investments in new technologies to support the optimal use of health care in current practice [29]. In our study, we adopted a welfarist economic approach and considered the cost-effectiveness threshold the shadow price of a marginal relaxation of the budget constraint.

Cost optimization through pharmacogenetic-guided treatments will permit the use of more efficient monitoring interventions for the management of patients taking antipsychotics, such as electrocardiographic screening to reduce drug-induced arrhythmias (i.e., Torsades-de-Pointe) and sudden cardiac death [30]. The rate of death from clozapine due to agranulocytosis was 0.1–0.2 per 1000 patient-years, which is much lower than the risk of sudden cardiac death in patients taking antipsychotic medications [31]. Furthermore, relieving the burden of the current ANCM will facilitate the use of clozapine, which has clinical and economic benefits for the treatment of TRS [32]. Stringent systematic requirements for weekly blood samplings can delay the initiation of clozapine and can impede patient recovery and unnecessarily prolong suffering.

According to our results, the pharmacogenetic testing was cost-effective up to a cost of $700 per patient. From a probabilistic approach, and given a WTP of $50 000 per QALY, there is a higher than 99% chance that HLA allele genotyping is cost-effective if HLA test costs remain under $700. This estimate is plausible because pharmacogenetic testing based on genotyping for the two aforementioned allelic variants of HLA-DQB1 and HLA-B (126Q and 158T) is a routine procedure in laboratories that test for HLA histocompatibility. Moreover, precise epitope determination could be established by designing adequate primers that can be used for simple PCR amplification at a very low cost.

The association of HLA alleles with an increased risk of agranulocytosis suggests an immune-mediated mechanism. The combination of genetic factors with clinical predictors of CIA, such as older age, sex, and the concomitant administration of other myelotoxic medications (e.g., propylthiouracil, azathioprine, metamizole) could help narrow the group of patients with HLA alleles of susceptibility for long-term ANCM. Although there is no convincing evidence of the direct toxicity of the parent compound or its stable metabolites (demethylclozapine and clozapine N-oxide), myeloid precursors are the affected target cells. Indeed, various HLA specific alleles have been demonstrated to be directly involved in drug toxicities, such as HLA-B*57:01 with the abacavir-induced hypersensitivity reaction [33], HLA-B*15:02 with carbamazepine-induced Stevens–Johnson syndrome, and toxic epidermal necrolysis with high test sensitivity [34].

One limitation of these results is the moderate performance of the genetic testing, as it remains a driving parameter for clinically effective testing in a small group with a higher CIA prevalence (1.7%). Because the test has relatively low sensitivity, it is hardly advisable to stop, without pilot studies or transition periods, ANCM in all TRS patients at the beginning of clozapine treatment. Furthermore, because of limited long-term data, our analysis could not extend beyond three years without strong assumptions. Finally, because we used a third-party payer perspective, we could neither incorporate unintended follow-up benefit and disutility (e.g., pain, practical, and time constraints) associated with ANCM nor intangible costs, such as productivity loss related to premature death.

The strengths of this study are the key parameters derived from the largest pharmacogenetic CIA study conducted to date. Additionally, the decision-analytical framework was based on a semi-Markovian model using a wide range of sensitivity analyses, which facilitated the probabilistic calculations of a single strategy and permitted transition between HS at any time during the treatment course in contrast with discrete-event models.

In conclusion, a pharmacogenetically guided strategy based on examinations of independent amino acid changes in both HLA-B (158T) and HLA-DQB1 (126Q) appeared to be cost-effective, allowing for the relaxation of long-term ANCM in low-risk patients taking clozapine despite the moderate sensitivity of these allele tests.

Similar analyses using less stringent monitoring schedules (e.g., the UK, Australia, Netherlands, and other European countries) should also be performed to assess the cost-effectiveness of pharmacogenetic-guided treatment and to calculate whether it is as economically worthwhile as in the US. Further research on HLA genome-wide genotyping and whole-exome sequencing will likely increase test performance and assist physicians in predicting agranulocytosis more efficiently, allowing a broader clinical application and supporting a review of the current monitoring schedules.

## Electronic supplementary material

Supplementary tables 1–3

Supplemental Figure Legend
