# Supplementary material for: Cost-effectiveness of HLA-DQB1/HLA-B pharmacogenetic-guided treatment and blood monitoring in US patients taking clozapine
Source: Pharmacogenomics J. 2018 Jan 3;19(2):211–8. doi: 10.1038/s41397-017-0004-2 (PMC6462824; doi:10.1038/s41397-017-0004-2)
Supplement: Supplementary file 1 — Supplementary tables 1–3 [file 41397_2017_4_MOESM1_ESM.docx]

**Supplementary Table 1: Input parameters**

|  | Base-case parameters and modeling | Probabilistic sensitivity analysis | Range for sensitivity analysis |
| --- | --- | --- | --- |
| State 1  Delay for Clozapine interruption | Weibull survival model (76% of interruption at 3 years) | Yes |  |
| Mortality under Clozapine | Exponential survival model (3-years mortality: 1.0%) | Yes |  |
| Risk of agranulocytosis | 0.7% |  | 0.38 – 2.0 |
| Delay for agranulocytosis | Regression model from individual data | Yes |  |
| State 2  Delay of infection occurrence since agranulocytosis | Exponential survival model (97% of infection at 14 days) |  |  |
| Infection-related mortality | 5% |  | 2.5 – 20.0 |
| State 3  Mortality for patients with treatment substitute | Exponential survival model (3-years mortality: 2.2%) | Yes |  |
| Costs  Clozapine treatment | $3.21 /day |  |  |
| Substitute treatment | $11.67 /day | Yes | 8 – 16 |
| ANCM screening | $31 / screening |  | 20 – 50 |
| Sepsis (hospitalization) | $31,398 / infection | Yes | 5,000 – 50,000 |
| Utilities  Patients with Clozapine (States 1 and 2) | 0.693 | Yes |  |
| Patients with substitute (State 3) | 0.560 | Yes |  |
| During the 10 days following an infection | 0.460 |  | 0.1 – 0.6 |
| Genetic test |  |  |  |
| Sensibility | 0.41 | Yes | 0.27 – 0.54 |
| Specificity | 0.85 | Yes | 0.80 – 0.90 |
| Cost | $200 | Yes | 0 – 1,000 |

The costs of hospital admissions for sepsis were calculated on the basis of the Swiss medical statistics of hospitals[[29](#_ENREF_29)] valued by Swiss DRG tariff rates[[30](#_ENREF_30)] and corrected for inflation between July 2012 and July 2013.[[31](#_ENREF_31)]

The cases of sepsis due to substance-induced neutropenia were identified by an ICD-10-GM main diagnosis of sepsis (A39.2, A40, A41, R65) or substance-induced neutropenia (D70.10-D70.19) together with a secondary diagnosis of the other without secondary diagnoses of cancer: fifty-one cases fulfilled these inclusion criteria in the whole MedStat database. The average length of stay was 19.4 days, and the average cost per inpatient stay was $31 398 for the year 2013; the cost calculations were consistent with the US Medical Fee Schedules, widely tested in the sensitivity analyses.

All cost results are reported in US$, with a rounded exchange rate of US$1.00 = 1.00 Swiss franc (March 20^th^, 2017).

**Supplementary** **Table 2: Parameter distribution**

|  | **Parameters / Modelling** | **Parameter distribution** |
| --- | --- | --- |
| Health state 1  Delay for clozapine interruption | Weibull survival model with  intercept=6.388 and log(scale)=0.549 | Intercept~ N (6.388, 0.005)  Log (scale) ~ N (0.549, 0.003) |
| Mortality under clozapine | Exponential survival model with rate λ=864 x 10^-8^ | λ ~ N (864.10^-8^, 617.10^-9^) |
| Delay for agranulocytosis | Delay was on average 48 days | Log (delay)~ N (3.866, 0.106) |
| Health state 3  Mortality for patients with treatment substitute | Piecewise exponential survival model with rates:  λ_1_=864.10^-8^ in (0-14) days  λ _2_=324.10^-6^ in (15-106) days  λ _3_=193.10^-7^ after 107 days | λ _1_~N (864 x 10^-8^, 617 x 10^-9^)  λ _2_~N (324 x 10^-6^, 344 x 10^-8^)  λ _3_~N (193 x 10^-7^, 183 x 10^-8^) |
| Costs |  |  |
| Substitute treatment | $10.57/day | N (10.57, 2.84) |
| Sepsis | $31 398 / infection | Gamma (0.59, 53342.28) |
| Utilities  Patients with clozapine (health states 1 + 2) | 0.693 | Beta (575, 255) |
| Patients with substitute (health state 3) | 0.560 | Beta (86, 67) |

We assigned beta distributions to utilities, gamma distributions to sepsis cost, normal distributions to other costs and to parameters of the exponential survival functions, and a multivariate normal distribution to the parameters of the Weibull survival model estimating time to clozapine discontinuation.

**Supplementary Table 3: One-way sensitivity analysis results (given a genetic testing cost of US$200**

|  |  |  | **ANCM strategies** | | |
| --- | --- | --- | --- | --- | --- |
| **Outcome** | **Parameters** | **Range** | **Current US** | **GGS** | **CSS** |
| Cumulative mortality (%) | Agranulocytosis rate (%) | 0.38 – 2.0 | 1.68 – 1.75 | 1.69 – 1.79 | 1.76 – 1.84 |
|  | Infection-related death rate (%) | 2.5 – 20.0 | 1.69 – 1.75 | 1.70 – 1.79 | 1.77 – 1.84 |
|  | Allele prevalence (%) | 10.0 – 20.0 | 1.70 – 1.70 | 1.71 – 1.71 | 1.76 – 1.80 |
| Survival time (days) | Agranulocytosis rate (%) | 0.38 – 2.0 | 1,086-1,086 | 1,086-1,085 | 1,086-1,085 |
|  | Infection-related death rate (%) | 2.5 – 20.0 | 1,086-1,085 | 1,086-1,085 | 1,086-1,085 |
|  | Allele prevalence (%) | 10.0 – 20.0 | 1,086-1,086 | 1,086-1,086 | 1,086-1,085 |
| QALYs (days) | QoL post infection | 0.1 – 0.6 | 670-670 | 670-670 | 660-660 |
|  | QoL in health states 1 and 2 | 0.56 – 0.9 | 608-766 | 608-766 | 608-742 |
|  | QoL in health state 3 | 0.46 – 0.69 | 608-751 | 608-751 | 591-750 |
|  | Agranulocytosis rate (%) | 0.38 – 2.0 | 670-668 | 670-668 | 661-658 |
|  | Infection-related death rate (%) | 2.5 – 20.0 | 670-670 | 670-669 | 660-660 |
|  | Allele prevalence (%) | 10.0 – 20.0 | 670-670 | 670-670 | 664-657 |
| Total cost (US$) | Cost of substitute | 8.0 – 16.0 | 11,016-19,460 | 10,413-18,855 | 10,805-20,053 |
|  | Cost of infection | 5,000 – 50,000 | 13,638-14,013 | 12,997-13,622 | 13,667-14,139 |
|  | Cost of a screening | 20.0– 50.0 | 13,332-14,319 | 13,036-13,185 | 13,738-13,738 |
|  | Agranulocytosis rate (%) | 0.38 – 2.0 | 13,610-14,035 | 12,973-13,570 | 13,644-14,120 |
|  | Infection-related death rate (%) | 2.5 – 20.0 | 13,698-13,670 | 13,098-13,051 | 13,743-13,708 |
|  | Allele prevalence (%) | 10.0 – 20.0 | 13,694-13,694 | 13,053-13,125 | 13,480-13,974 |
| ICER  (million US$ per QALY)* | Agranulocytosis rate (%) | 0.38 – 2.0 |  | 6.69 – 0.93 |  |
|  | Infection-related death rate (%) | 2.5 – 20.0 |  | 6.78 – 0.90 |  |
|  | Allele prevalence (%) | 10.0 – 20.0 |  | 3.24 – 4.88 |  |
|  | QoL in health states 1 and 2 | 0.56 – 0.9 |  | 3.84 – 4.08 |  |
|  | QoL in health state 3 | 0.46 – 0.69 |  | 5.10 – 2.98 |  |
|  | Infection rate† | 0.99 – 0.85 |  | 4.72 – 2.93 |  |
|  | Cost of substitute | 8.0 – 16.0 |  | 3.93 – 3.94 |  |

*The genotype-guided sampling (GGS) was the reference strategy. †The rate of infection within 14 days was used in the exponential survival model to estimate the delay of infection occurrence since agranulocytosis.

ANCM: Absolute neutrophil count monitoring, QoL: Quality of Life; QALY: Quality-adjusted life year; ICER: Incremental cost-effectiveness ratio.
