# Supplementary material for: Cost-effectiveness of HLA-DQB1/HLA-B pharmacogenetic-guided treatment and blood monitoring in US patients taking clozapine
Source: Pharmacogenomics J. 2018 Jan 3;19(2):211–8. doi: 10.1038/s41397-017-0004-2 (PMC6462824; doi:10.1038/s41397-017-0004-2)
Supplement: Supplementary file 2 — Supplemental Figure Legend [file 41397_2017_4_MOESM2_ESM.docx]

Supplementary Fig. 1| Cost-effectiveness plans with scatter plots.

Scatter plots of the CSS (A) and GGS (B) compared with the current ANCM scheme. Monte Carlo simulations output for the cost-effectiveness plan.

Most of the 10 000 simulations fell in the north-west quadrant, i.e., the more expensive and less effective quadrant, of the cost-effectiveness plan.

Most of the 10 000 simulations for GGS fell in the south-west quadrant, i.e., the less expensive and marginally less effective quadrant, compared to current US monitoring.
